# Supplementary material for: Collaborative intelligence in AI: Evaluating the performance of a council of AIs on the USMLE
Source: PLOS Digit Health. 2025 Oct 9;4(10):e0000787. doi: 10.1371/journal.pdig.0000787 (PMC12510544; doi:10.1371/journal.pdig.0000787)
Supplement: S2 File — (ZIP) [file pdig.0000787.s002.zip › S2 Data Raw data files containing unprocessed question inputs and LLM outputs including transcripts of deliberations/STEP3_Question-81-1_Type-MC-NJ.docx]

**Facilitator** **[Round 1]:** A 25-year-old woman comes to the office because of a 2-day history of right lower quadrant abdominal pain and vaginal spotting. She describes the abdominal pain as cramping and rates it as a 4 on a 10-point scale. Medical history is unremarkable and the patient takes no medications. Menses are typically irregular but she thinks her last menstrual period was approximately 5 weeks ago. She is sexually active with one male partner and they use condoms occasionally. Vital signs are temperature 37.2°C (99.0°F), pulse 90/min, respirations 16/min, and blood pressure 110/65 mm Hg. Abdominal examination discloses tenderness to palpation of the right lower quadrant. Pelvic examination discloses dark blood in the vaginal vault. Hematocrit is 36%. Urine pregnancy test is positive. Pelvic ultrasonography shows a thickened endometrial lining and no adnexal masses. Which of the following is the most appropriate next step in management? (A) Admission to the hospital for observation (B) Diagnostic laparoscopy (C) Dilatation and curettage (D) Follow-up pelvic ultrasonography in 1 week (E) Serial serum β-hCG concentrations

**AI_E:** (1) RESTATING THE QUESTION: A 25-year-old woman, with irregular menstrual cycle, sexual activity, and sporadic use of prophylactics, displays symptoms of cramping in the abdominal right lower quadrant with vaginal spotting over the course of two days. The constructed timeline of her menstrual cycle implies potential pregnancy, which is evidenced by a positive urine pregnancy test. An ultrasound confirms endometrial thickening but no growths in the adnexa. Given these data, it's necessary to identify the best next course of clinical action, considering admission for monitoring, diagnostic laparoscopy, curettage and dilatation, a follow-up ultrasound, or β-hCG concentration monitoring. (2) PLANNING: It appears to be an assumption that her case may be a possible ectopic pregnancy, as the given manifestations are typical for this. However, another perspective could be that she may be experiencing an early normal pregnancy with implantation bleeding. This latter assumption should be evaluated in terms of sequence and priority since an ectopic pregnancy is a life-threatening condition. (3) CLINICAL REASONING: Although ectopic pregnancies indeed manifest with lower abdominal pain and irregular bleeding, the absence of adnexal masses on ultrasound somewhat argues against this diagnosis. Normal pregnancies, however, can also present with these symptoms, but the ultrasound should display a gestational sac. The thickened endometrial lining may indicate recent implantation, hence the spotting. However, its absence could owe to the timing, as a gestational sac may not be visible within the first five weeks. (4) CONCLUDING: To resolve the questions surrounding the case, the quantitative measurement of serial serum β-hCG concentrations seems to be a fairly appropriate next step in management. In a healthy intrauterine pregnancy, β-hCG concentrations should approximately double every 48 hours. Values that increase less rapidly might suggest a less viable pregnancy, such as an ectopic one. (5) RESPONSE: Serial serum β-hCG concentrations (E) would allow for the monitoring of pregnancy vitality and viability. This information could then guide the subsequent course of clinical action.

**AI_B:** RESTATING THE QUESTION: A 25-year-old sexually active woman is experiencing lower abdominal pain and spotting. She has irregular menses and believes she had her last period approximately 5 weeks ago. Her medical history is clean, and she’s not on any medications. Her vital signs are normal. Upon physical examination, tenderness is observed in the lower right quadrant and dark blood in the vaginal vault. Her hematocrit level is 36%, and a urine pregnancy test returned positive. An ultrasonography showed a thickened endometrium but no adnexal masses. We are tasked with determining which course of action is most appropriate for her situation from the following options: hospital admission for observation, diagnostic laparoscopy, dilatation and curettage, a follow-up pelvic ultrasonography in one week, or serial serum β-hCG concentrations. PLANNING: To choose the best course of action, we need to take into consideration the woman's age, her symptoms, the irregularity of her menses, the positive urine pregnancy test, and the results of the pelvic ultrasonography. The primary assumption would be a normal early pregnancy given the positive pregnancy test and a thickened endometrial lining. However, the woman's abdominal pain and vaginal spotting are not usually present in early pregnancies. So, an alternate assumption could be an ectopic pregnancy or a failed intrauterine pregnancy (e.g., a miscarriage). Both are more serious conditions than an uncomplicated early pregnancy and should be prioritized clinically. CLINICAL REASONING: An ectopic pregnancy is a likely diagnosis given the woman's symptoms, but it's inconsistent with the ultrasound findings which show no adnexal masses. This rules out an ectopic pregnancy but introduces the possibility of a miscarriage. Therefore, it's important to monitor the levels of β-hCG—a hormone produced by the placenta during pregnancy. CONCLUDING: The most reasonable step would be to continually monitor the patient's levels of serum β-hCG rather than conducting a follow-up ultrasound. With a miscarriage, you would expect decreasing levels of β-hCG over time, while in a normal early pregnancy, β-hCG levels would be expected to rise every 48-72 hours. RESPONSE: Given the patient's positive pregnancy test, abdominal pain, vaginal bleeding, and ultrasound findings, the most appropriate next course of action would be to manage based on the assumptions of a likely miscarriage or normal early pregnancy. The best management here is obtaining serial serum β-hCG concentrations (E). This process will validate or disprove our assumption by observing whether levels of β-hCG decrease (indicative of a miscarriage) or increase (indicative of an early pregnancy).

**AI_D:** (1) RESTATING THE QUESTION: A 25-year-old woman with a history of irregular menstrual cycles and unprotected sex presents with lower right abdominal pain and spotting. The pain is described as a cramping sensation with a severity of 4 out of 10. She has a positive pregnancy test, and no evidence of adnexal masses on ultrasound. This scenario is suggestive of an early stage pregnancy, possibly complicated by concerns of an ectopic pregnancy or miscarriage. The question is asking which is the most appropriate course of action based on her signs, symptoms and test results. (2) PLANNING: The assumptions to consider are: 1) The abdominal pain and spotting could be early signs of a pregnancy complication such as ectopic pregnancy or spontaneous abortion; 2) The ultrasound findings are suggestive, but not definitive, of a possible complication; 3) The patient's menstrual history and use of occasional protection suggest a possible unintended pregnancy. Alternate assumptions could include that she might have a gastroenterological issue or a urinary tract infection, but the positive pregnancy test support assumption one. In terms of clinical priority, the likely pregnancy complication should be pursued. (3) CLINICAL REASONING: The clinical presentation of lower abdominal pain and bleeding, along with a positive pregnancy test, places the woman in a category of suspected ectopic pregnancy or threatened miscarriage. Despite the classic presentation of ectopic pregnancy, the ultrasound findings of no adnexal masses and a thickened endometrial lining are more suggestive of an intrauterine pregnancy, perhaps in some stage of abnormality. Given this, the priority is to monitor the progression of this pregnancy and ensure that a likely intrauterine process is safe and progressing accordingly. Thus, it would be necessary to monitor the serial serum β-hCG concentrations. (4) CONCLUDING: Following a clinical thought process, given the patient's clinical history, symptoms, and findings, this case is likely concerning for an abnormal intrauterine pregnancy since no adnexal masses were detected, but a thickened endometrial lining and pain may suggest an issue of potential for both ectopic pregnancy or intrauterine pregnancy with an issue. Since ectopic pregnancy can't be concluded, and given that she's stable, the best course of action would be to measure the changes in hCG to see if the pregnancy is viable or progressing normally. (5) RESPONSE: Thus, considering the information provided and the clinical thought process involved in ruling out ectopic pregnancy and following the development of what appears to be an intrauterine pregnancy, the most appropriate next step in managing this case would be to assess (E) Serial serum β-hCG concentrations.

**AI_C:** (1) RESTATING THE QUESTION: The patient is a 25-year-old woman with a 2-day history of abdominal pain, located in the right lower quadrant, and vaginal spotting. She reports her pain is cramping and rates it 4 out of 10. Her history is unremarkable and she does not take any medications. Although her periods are usually irregular, she estimates her last period was five weeks ago. She's sexually active with one partner, occasionally uses condoms for protection. Her vital signs are within normal range. Right lower quadrant is tender to touch. Dark blood is observed in her vaginal vault with a pelvic examination. She has a hematocrit of 36% and her urine pregnancy test is positive. Contrary to the expectation in early pregnancy, her ultrasound shows a thickened endometrium but no trace of adnexal masses. Which would be the most appropriate next step for her? (2) PLANNING: There are a few assumptions that one may have about her situation. One hypothesis might be that since she has a positive urine pregnancy test, she could be pregnant and this may be a tubal ectopic pregnancy, even though the ultrasound didn't reveal an adnexal mass. Another assumption could be that this is an early intrauterine pregnancy and she’s possibly experiencing a threatened abortion, again supported by her positive pregnancy test. Or perhaps she's experiencing an incomplete abortion. (3) CLINICAL REASONING: The current situation seems to favor the hypothesis of a pregnancy of unknown location (PUL). Her positive pregnancy test and thickened endometrium imply that she is pregnant. Yet, her ultrasound failed to identify intrauterine or ectopic pregnancy. This could be because the pregnancy is too early to be detected clearly by ultrasonography. A potential threatening condition is the possibility of an ectopic pregnancy, which could be life-threatening if untreated due to risk of rupture and bleeding. Despite not currently evident on ultrasound, early ectopic pregnancies can also give similar findings. Her right lower quadrant abdominal pain, along with a positive pregnancy test, creates a suspicious clinical picture for a possible ectopic scenario. (4) CONCLUDING: Based on these considerations, it becomes clear that the most reliable way to understand her situation better and ensure her safety would be to monitor the progress of her pregnancy. (5) RESPONSE: Serial serum β-hCG concentrations (E) would be the most appropriate next step. This would help confirm if the pregnancy is developing normally or abnormally (i.e., ectopic pregnancy). Typically, in normal intrauterine pregnancy, hCG levels double approximately every 48-72 hours in the early first trimester. Slower increases or decreases may indicate an abnormal pregnancy, such as an ectopic pregnancy or a miscarriage.

**AI_A:** RESTATING THE QUESTION: The inquiry revolves around a young woman who has been experiencing lower abdominal pain on the right side and vaginal spotting for two days. While her previous medical history raises no red flags, she does have irregular menstrual periods and was potentially pregnant five weeks ago. Her abdominal and pelvic examinations reveal that there's tenderness and bleeding, respectively. The pelvic ultrasonography has indicated a thickened endometrial lining. The key question here is what would be the best course of further action considering the current circumstances. PLANNING: The major assumptions in this case are that the woman has an ectopic pregnancy or a normal intrauterine pregnancy with some complications, given the positive pregnancy test. To generate further assumptions, we need to evaluate the known information. The vaginal bleeding and cramping abdominal pain suggest early pregnancy complications. The right lower quadrant pain and the positive pregnancy test might suggest ectopic pregnancy—an abnormal implantation of a fertilized ovum outside the uterine cavity. However, the absence of adnexal masses on ultrasound would contradict this assumption. The thickened endometrial lining could suggest an intrauterine pregnancy, but the presence of only a thickened endometrium and no clearly identifiable intrauterine pregnancy (i.e., gestational sac) on an ultrasound can also suggest a very early intrauterine pregnancy or a failed intrauterine pregnancy. Thus, the possibility of an ectopic pregnancy, an early normal or abnormal intrauterine pregnancy, needs to be considered, and decisive information is required to proceed further. CLINICAL REASONING: The ectopic pregnancy, although suspected, cannot be definitively ruled out simply by a negative ultrasound—such pregnancies can be overlooked on initial ultrasound in a significant proportion of cases. A growing ectopic pregnancy could also present as a thickened endometrium, simulating an intrauterine pregnancy, especially in the early stages. Hence, a possibility exists that this could be a pregnancy of unknown location, where you have a positive pregnancy test but no clear evidence of either intrauterine or ectopic pregnancy on ultrasound. In such cases, the standard protocol often involves serial serum β-hCG concentrations. β-hCG is a hormone that increases during normal early pregnancy. If the patient has an ectopic pregnancy, the β-hCG concentrations would typically rise slower or plateau, unlike in a normal intrauterine pregnancy, where it should roughly double approximately every 48 hours. CONCLUDING: It's important to find a balance line between being careful and not rushing a diagnosis that may potentially lead to unnecessary interventions. Considering the difficulty to locate a clear site of pregnancy clinically and ultrasonographically, the most reasonable further step in management, connecting all these factors, would be to monitor serial serum β-hCG concentrations to ascertain the progress and location of the pregnancy. RESPONSE: The most appropriate next step in management, therefore, in this patient's case is option (E), monitoring and comparing Serial serum β-hCG concentrations.

**Facilitator** The consensus among the AI responses is that the most appropriate next step in managing the case of the 25-year-old woman with a positive pregnancy test, lower right quadrant abdominal pain, and vaginal spotting is to monitor serial serum β-hCG concentrations. This decision is based on the woman's symptoms, her positive pregnancy test, and the ultrasound findings of a thickened endometrium but no adnexal masses. The primary assumption is that she could be experiencing an early pregnancy, possibly complicated by an ectopic pregnancy or miscarriage. However, the absence of adnexal masses on ultrasound somewhat contradicts the ectopic pregnancy assumption. Therefore, monitoring the β-hCG concentrations would help confirm if the pregnancy is developing normally or abnormally. In a healthy intrauterine pregnancy, β-hCG concentrations should approximately double every 48 hours. Values that increase less rapidly might suggest a less viable pregnancy, such as an ectopic one or a miscarriage. This approach would allow for the monitoring of pregnancy vitality and viability, guiding the subsequent course of clinical action.
